# New Opportunities to Mitigate the Burden of Disease Caused by Traffic Related Air Pollution: Antioxidant-Rich Diets and Supplements

**DOI:** 10.3390/ijerph17020630

**Published:** 2020-01-18

**Authors:** Jillian Barthelemy, Kristen Sanchez, Mark R. Miller, Haneen Khreis

**Affiliations:** 1 Center for Advancing Research in Transportation Emissions, Energy, and Health (CARTEEH), Texas A & M Transportation Institute (TTI), College Station, TX 77843, USA; jillianbarthelemy@tamu.edu (J.B.); k-sanchez@tti.tamu.edu (K.S.); 2 Centre for Cardiovascular Science, Queens Medical Research Institute, The University of Edinburgh, Edinburgh EH16 4TJ, UK; mark.miller@ed.ac.uk; 3 Barcelona Institute for Global Health (ISGlobal), Centre for Research in Environmental Epidemiology (CREAL), 08003 Barcelona, Spain

**Keywords:** reduce burden of disease, antioxidant supplement, antioxidant-rich diet, health effects, inflammation, Mediterranean diet, oxidative stress, traffic-related air pollution

## Abstract

Air pollution is associated with premature mortality and a wide spectrum of diseases. Traffic-related air pollution (TRAP) is one of the most concerning sources of air pollution for human exposure and health. Until TRAP levels can be significantly reduced on a global scale, there is a need for effective shorter-term strategies to prevent the adverse health effects of TRAP. A growing number of studies suggest that increasing antioxidant intake, through diet or supplementation, may reduce this burden of disease. In this paper, we conducted a non-systematic literature review to assess the available evidence on antioxidant-rich diets and antioxidant supplements as a strategy to mitigate adverse health effects of TRAP in human subjects. We identified 11 studies that fit our inclusion criteria; 3 of which investigated antioxidant-rich diets and 8 of which investigated antioxidant supplements. Overall, we found consistent evidence that dietary intake of antioxidants from adherence to the Mediterranean diet and increased fruit and vegetable consumption is effective in mitigating adverse health effects associated with TRAP. In contrast, antioxidant supplements, including fish oil, olive oil, and vitamin C and E supplements, presented conflicting evidence. Further research is needed to determine why antioxidant supplementation has limited efficacy and whether this relates to effective dose, supplement formulation, timing of administration, or population being studied. There is also a need to better ascertain if susceptible populations, such as children, the elderly, asthmatics and occupational workers consistently exposed to TRAP, should be recommended to increase their antioxidant intake to reduce their burden of disease. Policymakers should consider increasing populations’ antioxidant intake, through antioxidant-rich diets, as a relatively cheap and easy preventive measure to lower the burden of disease associated with TRAP.

## 1. Introduction

Ambient air pollution is the greatest environmental risk factor for human health, being associated with considerable levels of mortality and morbidity worldwide [1]. Globally, air pollution has been estimated to result in 4.2 million premature deaths annually [1]. Traffic-related air pollution (TRAP) is a prominent source of ambient air pollution, and one of particular concern for human health [2,3]. It is estimated that almost 20% of the United States population lives near a high traffic volume road, the exposure to which disproportionately affects minorities and those with low socioeconomic status [4]. A 2015 study using spatial mapping techniques revealed that 24% of the population in Toronto (Canada), 41% of the population in New Delhi (India), 66% of the population in Beijing (China), 67% of the population in Paris (France), and 96% of the population in Barcelona (Spain) are exposed to higher levels of TRAP when compared to background concentrations [5]. TRAP originates from motorized vehicles that generate exhaust and non-exhaust emissions. Exhaust emissions are produced from the combustion of fuel in motorized vehicles [6]. Non-exhaust emissions result from tire, brake, and road wear particles in addition to resuspended dust. The exhaust and non-exhaust emissions contribute to ambient air pollution both by primary emissions and formation of secondary pollutants [7]. Common traffic-related pollutants include particulate matter with a diameter of 10 micrometers or smaller (PM_10_), particulate matter with a diameter of 2.5 micrometers or smaller (PM_2.5_), ultrafine particles (UFPs), nitrogen oxides (NO_x_), nitrogen dioxide (NO_2_), carbon monoxide (CO), sulfur dioxide (SO_2_), black carbon (BC), elemental carbon (EC), hydrocarbons (HCs), and volatile organic compounds (VOCs) [8]. In 2013, traffic was responsible for 38% of NO_x_ emissions, 14% of VOC emissions, and 34% of all CO emissions in the United States [9]. NO_2_ and particles with smaller size ranges (e.g., UFPs) are hallmarks of traffic pollution, especially emissions from diesel exhaust. While disentangling the source of individual pollutants is challenging, BC and EC in particular, are commonly used as indicators of traffic in urban environments, given the high contribution of vehicles to combustion-derived emissions.

The prominence of TRAP exposures is a major public health concern as they have been associated with many adverse health effects. In general, air pollution is associated with cardiovascular diseases, respiratory diseases and premature mortality [10]. Notably, there is substantial evidence supporting an association between exposure to air pollution and premature mortality. The seminal Harvard Six Cities study demonstrated the association between ambient concentration of particulate matter (PM), ozone (O_3_), SO_2_, and sulfates (SO_4_) and loss of life [11]. More specific to TRAP, other studies have documented associations between living near a major road and cardiopulmonary mortality [12], long term exposures to air pollution and cardiovascular mortality [13], lung cancer mortality [14], and mortality from strokes [15]. TRAP has furthermore been linked to pulmonary morbidity, including onset of asthma and exacerbation of asthma symptoms in children [16,17,18,19], increased risk for persistent wheezing in children [20], decreased lung function [21] and the development of chronic obstructive pulmonary disease in adults (COPD) [21,22]. Other adverse health effects associated with common traffic-related air pollutants include diabetes [23], increased blood pressure [24], hospital admissions for circulatory diseases, myocardial infarction, lung cancer, kidney cancer, low respiratory tract infections [25], respiratory hospital admissions [26,27], decreased bone density and osteoporosis-related hospital visits [28]. More recent studies have demonstrated a link between traffic-related air pollutants and neurological effects and disorders [29,30]. For example, exposure to PM_10_ and NO_2_ in children was associated with the childhood attention hyper deficit hyperactivity disorder (ADHD) diagnosis [31], and children exposed to elemental carbon attributable to traffic (ECAT) had increased depression and anxiety symptoms [32]. Emerging research reveals an association between TRAP and declines in cognitive function [32,33], lower scores on a cognitive reasoning tests [34], lower verbal learning performances, lower logical memory abilities and lower executive functioning [35]. Dementia incidence has also been associated with increased exposures to PM_2.5_ [36], NO_x_ [37], NO_2_, and CO [30]. Furthermore, exposures to PM_10_ and O_3_ (which can be formed from the reaction of hydrocarbon emissions with sunlight) were associated with increased risk for Alzheimer’s disease and vascular dementia [38]. The widespread impact of TRAP warrants urgent strategies to mitigate its adverse health effects. 

There are many biological mechanisms by which different air pollutants can have adverse effects on the body. One prominent mechanism is oxidative stress: The formation of reactive oxygen species (ROS) beyond that which can be removed by cellular defenses, leading to the harm of cells and organs. Among ROS involved in oxidative stress are free radicals: forms of oxygen with unpaired electrons in the outer shell of their molecule, which lead to high levels of reactivity. Low levels of ROS play a natural role in cellular function by fine regulation of signaling and acting as a cellular defense mechanism for inflammatory cells where they are used to mediate inflammatory response and kill invading pathogens [39]. However, external stimuli, such as trauma, alcohol, medications, and exposure to radiation or toxins, can lead to an excessive amount of ROS, thus overcoming cellular defenses [40]. Air pollutants such as NO_x_, O_3_, PM_2.5_, and UFP are potent oxidants that are able to generate ROS and induce oxidative stress which can lead to cell death, inflammation, and injury [39,40]. Consequently, the promotion of oxidative stress has been identified as one of the most important mechanisms responsible for toxic air pollutant effects [41]. Fortunately, the body is equipped to handle a certain amount of oxidative stress. The term “antioxidants” refers to the biological chemicals and molecular systems that provide protection against oxidative stress. For example, the nasal cavity contains high levels of uric acid, a powerful antioxidant, and the lungs are lined with an extracellular antioxidant defense system comprised of reduced glutathione, ascorbic acid (vitamin C), uric acid, and alpha tocopherol (vitamin E) [40]. The excessive oxidative stress caused by pollutants can lead to the development of adverse health effects in the lungs and beyond, including decreased lung function, increased airway hyperactivity, pulmonary inflammation, damaged lungs and cell permeability [40]. Pulmonary effects can also progress to systemic effects through alterations in oxidative mediators and close interaction with inflammatory processes.

Several studies have investigated oxidative stress as a mechanism for the adverse health effects of air pollution. Oxidative stress can be quantified through a variety of methods, including measurement of ROS-modified molecules, indirect biomarkers of oxidative stress and alterations in levels of antioxidant activity. These biomarkers have been used to demonstrate that air pollution exposures are associated with oxidative stress and inflammation in humans [42], with support and further mechanistic insight provided by preclinical studies [43]. In this context, antioxidants represent a potential preventive strategy for disease processes due to their ability to blunt the effects of oxidative stress. The Food and Drug Administration (FDA) defines antioxidants as substances that, following absorption from the gastrointestinal tract, participate in physiological, biochemical, or cellular processes that prevent free radical-initiated chemical reactions or inactivate free radicals altogether [44]. Antioxidant provision can occur through diet or supplementation. Diets rich in soy, fruits, vegetables, nuts, seeds, oils, whole grains, some spices, and compounds found in chocolate and wine are great sources of antioxidants [45]. An example of an antioxidant-rich diet is the Mediterranean diet. The Mediterranean diet is rich in antioxidants due to common consumption of fruits, vegetables, whole grains, healthy fats, nuts, and fish [46]. Furthermore, antioxidants may be consumed as a supplement, which typically take the form of an oral pill. Once ingested, the antioxidants are absorbed by the gastrointestinal tract, leading to systemic availability [44]. Many antioxidant supplements are available, including vitamin C, vitamin E, beta-carotene, omega-3 polyunsaturated fatty acids (PUFA) and selenium [47].

A 2008 review addressed the potential of antioxidant diet and supplements for ameliorating the adverse effects of air pollution [48], and a more recent 2018 review discussed antioxidant use for the protection against the pulmonary effects of air pollution [49]. However, there is a need for an up-to-date literature review that holistically examines both antioxidant-rich diets and antioxidant supplements and for the various health outcomes associated with TRAP. The current review addressed this topic by compiling and analyzing the existing literature (until July 2019) on antioxidant-rich diets and supplements to mitigate adverse health effects of TRAP, in human subjects.

This paper is not meant to be a systematic review of the evidence but rather an overview of studies we identified through a structured and up-to-date literature search and expert knowledge. Time and resource constraints did not allow for the completion of a systematic review. However, this non-systematic review provides a critical assessment of some of the studies on antioxidant-rich diets and antioxidant supplements that may mitigate the burden of disease associated with TRAP exposures. It also paves the way for more informed future reviews and systematic reviews. Our aim is that this summation of the evidence will be of use to researchers, public health practitioners, clinicians, policymakers, and other stakeholders with an interest in the health effects of TRAP and how to mitigate them.

## 2. Materials and Methods

We searched the following databases and search engines: PubMed, Web of Science, Science Direct, Google and Google Scholar. We focused on literature published in the past 15 years by limiting the studies to only include those published between 1 January 2004 to 18 July 2019 (time of search). Relevant studies were identified using the following keywords and keyword combinations: “air pollution and health effects”, “health effects of air pollution”, “oxidative stress”, “oxidative stress and air pollution”, “antioxidants and air pollution”, “dietary intake and air pollution”, “Mediterranean diet and air pollution”, “dietary supplements and air pollution”, “dietary interventions and air pollution”, “antioxidant diet and air pollution”, “nutrition and air pollution”, “antioxidant polyunsaturated fatty acids”, “oxidative stress and antioxidant diet and air pollution”, “oxidative stress and antioxidant supplement and air pollution”, and “oxidative stress and antioxidant and air pollution”. Studies were exported to Mendeley reference manager software and any duplicates were removed. We also identified relevant reviews by expert knowledge.

It is worth noting that animal studies, which may have provided more evidence on antioxidants and underlying mechanisms, were excluded from this review. We decided to focus on research in human subjects due to time and resources limitations, but also because mechanistic evidence has been reviewed elsewhere [50,51], and is beyond our primary expertise.

We selected studies that met all the following criteria:

Were published peer-reviewed journal articles offering insight to the relationship between antioxidant-rich diet or antioxidant supplement interventions and the adverse health effects of TRAP, or markers thereof.

Any design in human populations including cohort studies and clinical studies with controlled exposures.

We excluded studies that were:

Animal studies

Cell culture studies

Papers without an English translation

Papers where full text was not available

Papers with pollutants or exposures not relevant to traffic

Journal articles were first screened by title and abstract by J.B. Irrelevant articles were excluded while potentially relevant articles were retained. Potentially relevant articles were then screened again by reading their full text. Once again, irrelevant articles were excluded, and the remaining articles were retained for inclusion in this review. The reference lists of all articles were checked for additional relevant articles for inclusion. J.B. extracted data from included articles based on a predetermined template designed by H.K. and J.B. The data elements extracted and documented included: Study name and reference, study design, objective of the study, country of origin, population details, sample size, air pollutant(s) examined, nutrient or supplement intervention, analysis methods, key findings and notes/gaps. Other important and necessary information was extracted from articles’ supplementary materials. Data extraction was done manually by J.B. and independently checked by two other authors (50% by K.S. and 100% by H.K.). A narrative synthesis was conducted in order to summarize and discuss the findings of the included studies.

## 3. Results

### 3.1. Overview of Results

The literature search yielded 11 studies that fit the inclusion criteria (Table 1). Three were cohort studies and 8 were randomized controlled trials (RCTs). Sample sizes in the cohort studies ranged from 208 to 548,845 subjects. Various age groups were studied: 1 of the cohort studies examined adults age 50 to 71 years old, another examined asthmatic children between the ages of 6 and 14 and the third cohort study examined infants who were between 11 and 23 months. Sample sizes in the RCTs ranged from 29 to 267 subjects. One investigated asthmatic children between the ages of 7 and 11 years old, 5 RCTs examined healthy adults over the age of 18, whereas two used a more elderly population (50 to 72 years or over 60 years). Of the 11 studies that fit the inclusion criteria, 3 cohort studies examined participants’ dietary patterns through a self-answered dietary assessment to score their fruit and vegetable intake or their Mediterranean diet adherence and 5 of the 11 studies examined the use of fish oil supplementation. Two of these 5 compared fish oil supplementation to olive oil supplementation. Fish oil is known to contain antioxidants and has beneficial action on several antioxidant/inflammatory pathways. Olive oil was used as a partial-control for fish oil, however, it should be noted that it contains oleic acid, which is a key component of the Mediterranean diet that possesses a degree of antioxidant and anti-inflammatory properties. Two of the 11 studies examined the use of sulforaphane through a broccoli sprout beverage. Finally, 1 study examined the use of a combined supplement of vitamin E and vitamin C. The included studies looked at the following air pollutants: PM_2.5_, PM_10_, concentrated ambient PM (CAP), NO_2_, O_3_, diesel exhaust particles (DEP) and benzene (Table 1). Of the 11 studies that fit the inclusion criteria, 4 were conducted in the United States, 4 in Mexico, 2 in China and 1 in Spain. Three studies addressed antioxidant-rich diets while 8 studies addressed antioxidant supplementation. In the following sections, we describe the evidence from the 11 included studies under key categories of antioxidant-rich diets and antioxidant supplementations.

### 3.2. Fruit and Vegetable Rich Diet

Fruits and vegetables are well known for being rich in nutrients and antioxidants. In a 2012 birth cohort study, Guxens et al. [52] analyzed the effects of antioxidants on infant mental development and its relationship to prenatal exposure to NO_2_ and benzene. Prenatal exposure to air pollution was assessed by passive samplers distributed over the study areas and land use regression models were developed based on these measurements to predict average outdoor air pollution levels for the entire pregnancy at each residential address. Mothers used a self-reported food frequency questionnaire (focused on fruit and vegetable intake) during their first trimester and whether the infant was breastfed through the second year of life. A single maternal fasting blood specimen was drawn during pregnancy (mean ± SD, 13.4 ± 1.7 weeks of gestation) and maternal plasma vitamin D levels were determined. Mental development in infants between 11 and 23 months was assessed using the Bayley Scales of Infant Development. The Bayley Scales of Infant Development is composed of 163 items that assess age-appropriate mental development, performance abilities, memory, and early language skills. The results demonstrated an inverse association between both NO_2_ and benzene exposure with mental development. The inverse association between pollutants and mental development was also found in infants who were not breastfed. Neither of these results were statistically significant. However, the study did result in a statistically significant inverse relationship between pollutants and mental development among infants with low maternal intake of fruits and vegetables. This study suggested that the antioxidants consumed from fruits and vegetables may inhibit the cognitive impairments in infants that might have resulted from maternal exposure to air pollutants, especially in mothers who have a low antioxidant intake.

### 3.3. Mediterranean Diet

The Mediterranean diet is rich in antioxidants because it is rich in plant-based foods, such as whole grains, fruits and vegetables, and olive oil [62]. It also includes low consumption of meat, moderate alcohol consumption (generally in the form of wine) and fish [63]. This diet is associated with improved cardiovascular health, reduced inflammation and reduced oxidative stress responses [64]. A large cohort study conducted in the United States had 548,845 adults between the ages of 50 and 71 used a self-reported dietary questionnaire to formulate an alternative Mediterranean diet (aMED) score based on their dietary patterns [46]. Participants who reported greater adherence to the Mediterranean diet (i.e., had a high aMED score) had significantly lower rates of cardiovascular disease mortality associated with long-term exposure to NO_2_ and PM_2.5_, where long term exposure to air pollutants was determined as annual average concentration levels from 1994 to 2010. This supported the idea that greater adherence to the antioxidant-rich Mediterranean diet may blunt the adverse cardiovascular effects from air pollution exposure. Similarly, a dynamic panel cohort study of 208 children in Mexico City analyzed the relationship between fruit and vegetable intake (FVI) and Mediterranean diet index (MDI) with lung function and airway inflammation. Readings from the closest fixed site monitoring station were assigned to the child’s residential address and used to estimate their exposure to PM_2.5_, NO_2_, and O_3_. Children were followed for 22 weeks, with their pulmonary function measured every 2 weeks, and their nasal lavage was collected and analyzed for inflammatory markers. Asthmatic children with a higher FVI scores had statistically significant lower interleukin-8 (a mediator of inflammation, IL-8) in their lavage after exposure to PM_2.5_, NO_2_, and O_3_. Children who had high FVI scores had 8% lower IL-8 than children with low FVI scores. Furthermore, asthmatic children who had higher MDI scores had significantly higher lung function after exposure to PM_2.5_, NO_2_, and O_3_ [53]. Children with the highest MDI scores had a 15.3% higher forced expiratory volume in one second and 16.5% higher forced vital capacity than children with lower MDI scores, which indicates increased lung functioning in those with higher MDI scores [53]. However, no statistically significant protection from inflammation and reductions in lung function were found in the non-asthmatic children.

### 3.4. Fish Oil and Other Oil Supplements

Fish oil and (to a lesser extent) soy oil have anti-inflammatory effects and have been shown to reduce ROS generation and mitigate the oxidative stress response from stimuli, including air pollution [65]. In a randomized, double-blinded, controlled trial, the effects of fish oil were compared to the effects of soy oil in 50 nursing home residents (all >60 years old) who had been exposed to elevated levels of PM_2.5_ from the ambient air. The participants spent 93% of their time indoors, and the mean levels of ambient PM_2.5_ in the room where the study was conducted was 18.6 µg/m^3^ (24 h average). This study was in Mexico City, where the major source of PM_2.5_ pollution is vehicular traffic and a large proportion of vehicles use diesel fuel. The mean levels of ambient PM_2.5_ outdoors during the study period was 19.6 µg/m^3^ (24 h average). Participants were given either 2 g of soy oil per day (control) or 2 g of fish oil per day with a 6-month monitoring period (1-month pre-supplementation and 5 months supplementation). In the pre-supplementation phase, the group receiving fish oil supplements experienced a 54% reduction in parameters of heart rate variability (HRV); high frequency log10-transformed HRV associated with a 1 standard deviation change in PM_2.5_ exposure. During the supplementation phase, this reduction was 7%. Participants who were given 2 g of fish oil supplements per day experienced a statistically significant reduction in their heart rate variability (HRV) decline after being exposed to PM_2.5_. On the other hand, participants who were given soy oil supplements experienced marginal and non-significant changes in HRV after PM_2.5_ exposure [54]. Decreased HRV has been associated with increased mortality from sudden death and ventricular arrhythmia in both healthy and diseased individuals [57], thus the fish oil supplement suggest a beneficial effect whereas the soy oil did not.

In another RCT conducted by the same authors [55], 52 participants from a nursing home were given similar supplements of fish oil or soy oil for 4 months. Participants spent 93% of their time indoors where they were chronically exposed to ambient PM_2.5_ with a mean daily concentration of 38.7 µg/m^3^. Both fish oil and soy oil groups increased plasma levels of superoxide dismutase (SOD) activity and increases in levels of reduced glutathione (GSH) demonstrating systemic efficacy of the antioxidant supplements (both SOD and GSH are key antioxidant mediators). Additionally, the fish oil, but not the soy oil, group showed decreases in levels of lipoperoxidation (LPO), a marker of oxidative stress. A similar randomized, double-blinded, placebo-controlled trial in China analyzed the extent to which fish oil supplements (2.5 g per day) would protect against cardiovascular damage as a result of PM_2.5_ exposures. The participants were 65 healthy, college students (20 to 25 years old) who underwent a 5-month supplementation phase with 4 rounds of follow up visits with an interval of 2 weeks in the last 2 months of the intervention. In this study, the placebo group was given sunflower seed oil. There was greater levels of oxidative stress (higher levels of ox-LDL and lower activities of antioxidant capacity), inflammation and coagulation biomarkers, as well as endothelial dysfunction (higher levels of endothelin-1 and E-selectin, and lower levels of concentration of serum eNOS protein), and neuroendocrine disturbance in the placebo group compared to the fish oil supplement group [56].

Another RCT conducted in the United States found that healthy individuals (20 participants aged 50 to 72) given fish oil supplements (3 g per day) for 4 weeks prior to sequential 2 h chamber exposures to CAP and ultrafine particulate matter (mean: 278 ± 19 µg/m^3^), were protected from autonomic, cardiac electrophysiological, and lipid changes [57]. Cardiac electrophysiological changes were measured through HRV and cardiac repolarization while lipid levels and blood cells were analyzed to indicate lipid changes. Participants who received a similar olive oil supplement were unprotected from cardiac electrophysiological and lipid changes. Fish oil supplementation attenuated CAP-induced reductions in high frequency/low frequency ratio and elevations in normalized low-frequency HRV. Furthermore, participants who consumed olive oil supplements experienced significant increases in their normalized low-frequency HRV immediately after exposure to CAP, which persisted for at least 20 h, while those given the fish oil supplements did not experience any significant alteration in HRV in response to CAP exposures [57]. Furthermore, one RCT investigated the effect of olive oil compared to fish oil in mitigating endothelial dysfunction (a marker and risk factor for cardiovascular disease) and coagulation markers in response to a two-hour controlled exposure to CAP (mean: 253 ± 16 µg/m^3^, by drawing ambient air from above the roof and passing it through a 2-stage aerosol Harvard concentrator that produces up to a 30-fold increase in particle number and mass) in 42 middle-aged volunteers. Participants who consumed 3 g of olive oil per day for 28 days experienced a statistically significant increase in flow mediated dilation and increases in fibrinolysis blood markers which indicate a protective effect to attenuate vascular effects of exposure to CAP. This effect was not seen in participants consuming 3 g of fish oil per day [58]. These results warrant further investigation because other studies found that fish oil supplements provided protection from air pollutants. 

### 3.5. Sulforaphane

Sulforaphane is a molecule often found in cruciferous vegetables, such as broccoli, that has anti-inflammatory and antioxidant properties [66]. Due to its antioxidant nature, sulforaphane has been investigated for its ability to mitigate the adverse health effects of TRAP. Previous research conducted to investigate the effects of sulforaphane on the expression of oxidative stress genes revealed that sulforaphane had detoxification properties that could provide protection from the harm brought on by air pollutants [67].

A single blinded, placebo-controlled trial was conducted to analyze the effects of standardized broccoli sprout extract on nasal inflammatory response after exposure to DEP. DEP exposures of 300 µg in an aqueous suspension were administered from a dilution tunnel constant volume sampler. A standardized dose of 100 micromolar sulforaphane broccoli sprout extract in mango juice was given to 29 healthy adult participants for four consecutive days. Participants consuming the juice exhibited a lower inflammatory response (white blood cell counts) to the DEP compared to the control. [59]. Another randomized, placebo-controlled trial in China investigated whether daily consumption of a broccoli sprout beverage for 12 weeks could ameliorate the detrimental effects of benzene exposure. Participants who received the broccoli sprout beverage had a statistically significant higher urinary S-phenyl mercapturic acid (SPMA) and hydroxypropyl mercapturic acid (3-HPMA) excretion [60]. SPMA and 3-HPMA are markers for exposure to benzene air pollution because they are levels of mercapturic acids formed in the metabolism of benzene. These levels increase as a result of benzene exposure. Increased level of excretion of SPMA and 3-HPMA indicates that the broccoli sprout beverage enhanced detoxification of pollutants. While the broccoli sprout beverages prove to be useful in mitigating the adverse effects associated with the exposures to DEP and benzene, participants in the studies often complained of the beverage’s bad taste, and some experienced nausea from the drink.

### 3.6. Vitamin C and Vitamin E

Vitamin C and vitamin E have been the focus of several studies due to being inexpensive antioxidants that are widely taken as supplements across the world. In a randomized, double blinded, placebo-controlled trial conducted in Mexico, asthmatic children (7 and 11 years) were given a supplement comprising of 50 mg vitamin E and 250 mg vitamin C with measurements of nasal lavages were taken 3 times during a 4 month follow up and analyzed for content of interleukin-6, IL-8, uric acid, and glutathione. Children who received the placebo had statistically significant increases in interleukin-6 (IL-6; a marker of inflammation) in nasal lavage that was associated with O_3_ and PM_10_ exposure, while children receiving the antioxidant supplement did not [61]. However, the study did not account for information regarding allergens, which limits the results. 

## 4. Discussion

### 4.1. Summary and Key Findings

This literature review provides evidence that antioxidant-rich diets and antioxidant supplements can protect against the adverse health effects associated with exposure to air pollution, and specifically, TRAP. The evidence is largely consistent and originates from RCTs with relatively small numbers of volunteers and a limited number of population-based cohort studies. The data suggests that increased antioxidant intake, whether it be through diet or supplementation, can blunt the adverse health effects of exposure to common traffic-related air pollutants. However, the number of studies investigating this question is rather small (11) and further research is required to replicate and confirm previous findings.

Antioxidant-rich diets, such as the Mediterranean diet and increased fruit and vegetable intake, gave the most promising evidence for protection from TRAP. On the other hand, the benefits of olive oil, a feature of the Mediterranean diet, were inconsistent between studies. These results warrant further research in order to determine the effectiveness of olive oil in mitigating adverse health effects from TRAP exposure. In contrast, fish oil supplements provided more consistently effective results in mitigating the effects of TRAP. Fish oil supplements are relatively cheap and can be accessed easily without prescription. In addition to taking fish oil supplements, people could also increase their intake through diet by consuming fish rich in oils (n-3-PUFA). Fish that are rich in n-3-PUFA include sockeye salmon, farmed trout and salmon, Copper River salmon, Coho salmon, bronzini, and toothfish [68]. The effective dose of fish oil (in diet or as supplements) requires further research. Broccoli sprout beverages containing sulforaphane also mitigated the adverse health effects of TRAP exposure, albeit only two studies that were identified. However, people may be unlikely to consume a broccoli sprout beverage regularly if they dislike the taste or experience side effects such as mild gastrointestinal upset [59]. Finally, vitamin C and vitamin E were investigated in one study and were found to reduce inflammation associated with air pollution exposure. While the evidence suggests that vitamin C and E supplements may be useful in providing protection from TRAP, the overall findings of large clinical trials of these antioxidants assessing mortality and clinically-relevant end-points have been disappointing [69]. Concerningly, a meta-analysis of 19 clinical trials using vitamin E supplements found that high doses (≥400 IU/d for at least 1 year) of vitamin E may even increase all-cause mortality [70]. Vitamin C and E should therefore not be taken in high doses and consumed with precaution until there is more research to determine their effective dose to protect from pollutants. For all anti-oxidants identified in the literature, more research is needed to determine their appropriate dosages. Once the effective dose for each of these supplements is determined, they could offer a relatively cheap and accessible method to increase antioxidant intake, thus increasing protection from TRAP. Other factors to be considered when investigating the effectiveness of antioxidant supplementation would be the time the antioxidant is consumed, duration of supplementation, the balance between diet and supplementation, the amount of antioxidants that reach the bloodstream and whether or not antioxidants reach the areas of disease or subcellular locations. Further points of interest include what formulations can be used to increase bioavailability of antioxidants, whether formulations can be used to target specific organs or diseases, whether antioxidants combat only some parts of the disease pathways and not others, if are antioxidants useful for specific patient subgroups and not others, and finally, whether combinations of antioxidants needed to fully target multiple free radicals and disease processes.

Based on the results of this review, increasing people’s fruit and vegetable intake as well as encouraging adherence to the Mediterranean diet seem to be the most promising strategy. In addition to offering protection against TRAP effects, dietary interventions are associated with other co-benefits, such as reduced mortality and reduced risk of diseases overall by decreasing the inflammatory response associated with exposure and mitigating oxidant damage to cell structures [69]. Furthermore, dietary changes that increase antioxidant intake should be encouraged for all people, and especially for susceptible populations such as children, the elderly, asthmatics and those who are occupationally exposed to TRAP. Those who suffer from preexisting chronic conditions and chronic inflammation [48] and those who have damaged or weakened antioxidant defense systems may be more susceptible to oxidative stress [71]. Children are also a susceptible population because their organ systems are still developing, and therefore there is a greater cellular susceptibility to oxidative stress. Likewise, elderly persons have an increased susceptibility due to weakened immune and detoxification systems. Transportation workers, taxi drivers, construction workers, and street vendors may experience greater exposures to TRAP due to their occupational location and/or type of work. Indeed, a study revealed that taxi drivers had higher levels of oxidative stress biomarkers, increases in pro-inflammatory mediators and an increased risk for cardiovascular events, compared to non-occupationally exposed persons [72]. Genetic abnormalities can also engender greater susceptibility to more oxidative stress [73]. Polymorphisms in genes involved in oxidative stress such as NAD(P)Quinone oxidoreductase 1 (NQO1) and glutathione-S-transferase (GSTM1 or GSTP1) have been found to alter response to pollutants. For example, genetic variations in these genes led to a greater nasal inflammation, increased airway epithelial damage, and higher levels of oxidative stress in response to O_3_ exposure [73]. It is worth noting that 5 of the 11 articles included in this review investigated populations of potential susceptibility. Two RCTs enrolled elderly participants, 1 RCT enrolled asthmatic children, 1 cohort study investigated children, and 1 considered prenatal exposures of infants.

### 4.2. Comparison with Previous Studies

The findings of this literature review are consistent with findings of previous relevant studies. For instance, a RCT in 80 adults in Brazil investigated the effect of antioxidant supplementation (6 month of 500 mg of vitamin C and 800 mg of vitamin E) on biomarkers of oxidative stress linked to PM exposure originating from coal combustion emissions [74]. While this study is specifically not a TRAP exposure, there are similarities between the health effects of both these combustion-derived emissions and their pathways of action. Level of exposure was determined by whether people were directly exposed (those who handle the mineral coal), indirectly exposed (office workers of the electric power plant 200 m from the burning area), residents (subjects living in the city 2 kilometers from the burning area) or non-exposed subjects (controls who lived 100 km from the emissions). After taking the antioxidant supplement for 6 months, participants in all groups who were exposed to PM emissions (direct exposure, indirect exposure, and residents) had similar levels of biomarkers of oxidative stress as the control group [74]. 

Older studies before time frame for this literature review’s search criteria also present evidence that antioxidant supplementation can mitigate the adverse health effects of air pollution. A controlled trial in the Netherlands, investigated the use of a supplement (650 mg of vitamin C, 75 mg of vitamin E and 15 mg of beta carotene) in 26 young amateur cyclists exposed to O_3_. The cyclists who took the supplement experienced no effect from the O_3_ exposure on lung function (forced expiratory volume in one second, forced vital capacity, peak expiratory flow, and maximal mid expiratory flow) while the control group that received a placebo had adverse responses to O_3_ [75]. A double-blinded randomized trial in 158 children with asthma in Mexico City investigated the use of daily supplementation (50 mg of vitamin E and 250 mg of vitamin C). This study revealed that daily antioxidant supplementation provided protection against exacerbation of asthma symptoms from O_3_ and NO_2_ exposures [76]. Both studies provide more evidence supporting the claim that vitamin E and vitamin C can mitigate pulmonary decrements after exposure to traffic-related air pollutants and secondary pollutants (e.g., O_3_ which is promoted by traffic-related emissions).

### 4.3. Limitations

We did not intend this literature review to be an all-inclusive review of antioxidant interventions studied in relation to air pollution. Instead, we focused on human studies in the last 15 years, to provide a summation of recent findings that have relevance to TRAP and highlight key research findings and gaps from the most up-to-date evidence base. We acknowledge that the current review was non-systematic, searching specific databases and being limited to the last 15 years. Conducting a systematic review is likely to identify further evidence and potentially different conclusions. Furthermore, there is a substantial body of preclinical data from animals and cellular models that could provide useful mechanistic insight into the human observations. 

Another potential limitation of this review stems from the limitations of the studies analyzed and the methodologies employed in the included papers. For example, RCTs are not representative of the combination of TRAP exposures people experience in the real-world. Instead, they are usually limited to specific pollutants being tested in each experiment through controlled exposures. On the other hand, there are many benefits of controlled exposure studies over real-world investigations due to their ability to control exposures at specific rates over specific time periods and the minimization of bias, especially selection bias and confounding. Another limitation relates to the exposure assessment in the included studies. Often, exposure assessment was based on a fixed monitoring network rather than a personal monitor, which could lead to exposure misclassification. The increased availability of more accurate lower-cost personal sensors will allow gathering of more accurate individual exposures, or at the very least offer insight into the potential uncertainty arising from use of fixed monitoring data. The literature identified considered studies conducted in different geographic areas, which provides some benefits in terms of ascertaining the generalization of the observed effects in different populations. However, it also introduces other factors that could be confounders (e.g., different air pollution mixtures in different regions, genetic background, socioeconomic variables, differences in diet, and compliance for taking supplements). Finally, in several of the antioxidant supplement studies, some aspects of participants’ diets were not controlled for or only adjusted for at baseline, with no tracking of changes over the follow-up period. 

### 4.4. Research Recommendations

This short review raises the need for a systematic review of literature on this topic, however, it also allows us to make recommendations for practical research topics. We recommend that future studies specifically investigate the effects of antioxidants on vulnerable populations, such as children with asthma, the elderly, certain occupational workers and people with genetic susceptibility as these subpopulations may benefit the most from targeted dietary or supplemental interventions. Although several of the studies identified did focus on susceptible populations, it would be beneficial to expand the literature regarding those who experience increased responses to TRAP and specifically investigate genetic susceptibilities possibly in different ethnic populations. In the included studies, many participants were generally healthy adults. While healthy adults are also at risk of the adverse effects of TRAP, young and healthy adults may be more likely to consume better foods and be more active than the general population, which could influence exposure and response to pollutants and antioxidants [46]. Furthermore, this review warrants more detailed research regarding the role that obesity may play in the adverse health effects associated with TRAP. Obesity continues to be a major threat to health and contributes to other TRAP health effects listed in this review, such as asthma [77]. A vulnerable population that has not been discussed in the literature is lower socioeconomic status individuals. These populations exhibit a variety of factors which might heighten their risk of adverse health effects from TRAP exposures, including exposure to violence, stress, reduced access to health care and poor diet [78]. Additionally, there is a need for further investigation on the effective dose of antioxidant supplements, as well as a greater insight into reasons for conflicting results regarding the effectiveness of different antioxidant diets (e.g., fish oil and olive oil). Future research would also benefit from control for confounding effects from daily dietary intakes and changes in diet over follow-up. It should be noted that curcumin, an Asian spice that has antioxidant properties, was not included in this review. A meta-analysis suggests that the antioxidant properties of curcumin could mitigate depressive and anxiety symptoms in patients [79]. Therefore, future research could be aimed at investigating the potential for curcumin to mitigate the burden of disease caused by TRAP. Finally, more research could be undertaken to evaluate the potential of antioxidants in mitigating air pollution effects in other organs or diseases of the body than were discussed in this paper.

### 4.5. Policy Recommendations

Policy makers should continue to pursue legislation that would reduce levels of TRAP. In 2016, 91% of the world’s population still lived in areas that exceeded the World Health Organization air quality guideline values [80], which are even too high to fully protect public health. However, until the long-term goal of cleaning the air can be met, policy makers could consider the use of antioxidant-rich diets, and to a lesser extent supplements, as a means of mitigating adverse health effects associated with TRAP. For example, schools in highly polluted areas may be incentivized to serve lunches that are antioxidant rich. Simple changes to a school lunch menu could be serving oil rich and organic fish at least once a week and increasing the organic fruits and vegetables at every meal. Furthermore, nursing homes and other care facilities for older adults should consider employing the use of antioxidant-rich diets. In addition to the Mediterranean diet reducing inflammation and mitigating adverse health effects of TRAP, there is evidence that the Mediterranean diet supplemented with extra virgin olive oil and mixed nuts can reduce age related cognitive decline in the elderly [81]. Such evidence might be applicable to the increasing body of evidence showing that air pollution, and TRAP specifically, is linked to cognitive decline [33,34,35,82]. It is important to address TRAP in particular as vehicles are one of the major sources of ambient air pollution, which specifically act in close proximity to an increasing proportion of people, thus making the adverse impacts of TRAP exposure widespread [78,80].

### 4.6. Practice Recommendations

Clinicians and health practitioners should encourage their clients to consume an antioxidant-rich diet and increase their organic fruit and vegetable intake, especially for children, elderly, pregnant women, and asthmatics. A cost-effective population-based intervention would be to bring awareness to the importance of dietary practices. Furthermore, occupations that are chronically exposed to TRAP, such as construction workers, transportation workers, and street vendors, should recommend or incentivize their employees to increase their nutritional or supplemental antioxidant intake. Raising awareness in dietary health benefits can be provided relatively easily, for little cost and in a manner that goes hand-in-hand with improving education for all on the health risks of air pollution.

## 5. Conclusions

Antioxidants may reduce the effects of oxidative stress because they can remove oxidizing agents and inhibit oxidation. Because of this, an antioxidant-rich diet or antioxidant supplement intake can potentially be used as a preventive strategy for the harmful health effects of TRAP. In this literature review, we identified 11 papers in the last 15 years that considered the potential benefits of increased antioxidant intake through diet or supplementation on mitigating the adverse health effects of exposure to common traffic-related air pollutants. Antioxidant interventions investigated ranged from adherence to the Mediterranean diet, increased fruit and vegetable intake, consumption of fish oil and olive oil, sulforaphane intake via broccoli sprout beverages, and vitamin C and E supplements. Major points to note from the literature include fruit and vegetable rich diets, the Mediterranean diet, fish oils and vitamin C and vitamin E provide protection from the adverse health effects associated with TRAP. Greater adherence to a diet rich in fruits and vegetables or the Mediterranean diet was effective in reducing the adverse health effects associated with TRAP and had no negative effects associated with them. While there are inevitably inconsistencies in the findings, the overall weight of evidence suggest that antioxidant intake can ameliorate the effects of TRAP in different organ systems. There is good evidence to recommend that healthy and susceptible individuals should practice greater adherence to the Mediterranean diet and increase their fruit and vegetable intake. There is conflicting evidence regarding the use of antioxidant supplements and thus a need for more research focusing on antioxidant supplementation. Future research should focus on conducting cohort studies to analyze long-term effectiveness of antioxidants in studies that are representative of real-world exposures and with better control for confounders. Given the near ubiquitous exposure to pollution worldwide, and the huge impact on health, these findings have relevance to the general public, but also to health practitioners, clinicians, employers and policymakers seeking to limit the burden of air pollution on health. Specifically, susceptible populations including asthmatics, elderly and those with genetic variations should increase their antioxidant intake in order to attenuate the adverse health effects associated with air pollution and TRAP exposures. In conclusion, until lower levels of TRAP are achieved, antioxidant-rich diets and supplementation may offer promise for reducing the burden of disease associated with TRAP exposures.

## Figures and Tables

**Table 1:** Characteristics of the included studies.

Study Name and Reference	Study Design	Objective	Country of Origin	Population	Sample Size	Air Pollutants	Nutrient Intervention	Analysis Methods	Key Findings	Notes/Gaps
**Fruit and Vegetable Rich Diet**										
Guxens et al., 2012 [52]	Birth cohort study	Analyze the effect of antioxidants and detoxification factors on infant mental development that results from prenatal exposure to air pollution.	Spain	Infants (11 to 23 months of age at time of assessment)	1889	NO_2_ and benzene were measured with passive samplers distributed over the study areas and land use regression models were developed based on these measurements to predict average outdoor air pollution levels for the entire pregnancy at each residential address.	Mothers were given a validated food frequency questionnaire during their first trimester, focusing on fruit and vegetable intake. Information on maternal diet was obtained using a 101-item semi-quantitative validated food frequency questionnaire. Detailed information about child feeding through the second year of life was collected from mothers by interviewer-administered questionnaires. A single maternal fasting blood specimen was also drawn during pregnancy and levels of maternal plasma vitamin D were determined.	Multivariable linear regression models were performed to examine the relationship between log-transformed NO_2_ and benzene and infant mental development as continuous normal variables. A meta-analysis using random-effects models combining the estimates in each region of the association between each air pollutant and infant mental development was conducted. Effect modification of air pollution associations by levels of antioxidants and detoxification factors (fruits and vegetables, breast-feeding, circulating vitamin D), as well as by socioeconomic variables (parental social class and maternal education), was assessed using stratified analysis and interaction terms. The analysis adjusted for psychologist, child’s sex, child’s age at mental development assessment, maternal education, maternal age, maternal height and pre-pregnancy body mass index, maternal alcohol use during pregnancy, maternal large fatty and lean fish consumption at first trimester, season-specific maternal circulating vitamin D level at pregnancy, use of gas stove at home during pregnancy, and number of siblings at birth.	Mental development was assessed using the Bayley Scales of Infant Development. The Bayley Scales of Infant Development is composed of 163 items that assess age-appropriate mental development, performance abilities, memory, and early language skills. There was an inverse relationship between NO_2_ and benzene exposures and mental development, however, it was not statistically significant after adjusting for potential confounders. A statistically significant inverse relationship between pollutants and mental development was limited to infants with low maternal intake of fruits and vegetables during pregnancy. Inverse associations between pollutants and mental development in infants who were not breastfed and infants with low maternal vitamin D were also observed, however, these effect estimates, and interactions were not statistically significant.	Breast milk has long chains of polyunsaturated fatty acids and other micronutrients that reduce inflammation and oxidative stress. The main limitations reported by the authors were that noise annoyance during pregnancy was collected by a self-reported scale rather than by a direct measure of noise levels (and was used in sensitivity analysis). As such, residual confounding from noise may remain. Parental intelligence, an important determinant of infant mental development, was not evaluated although parental education and social class were but did not confound or modify the associations. Finally, not all children initially recruited at birth were included in the final analysis, and loss due to follow-up (25%) was related to lower social status, although this variable was adjusted for.
**Mediterranean Diet**										
Lim et al., 2019 [46]	Prospective cohort study	Analyze the potential effect of a Mediterranean diet on the relationship between air pollutants and cardiovascular disease mortality risk.	United States	Adults (ages 50 to 71)	548,845	PM_2.5_, NO_2_ were modeled using a spatiotemporal prediction model and a recent model, which applied kriging models combining land-use regression methods with satellite data, respectively.	Mediterranean diet, which emphasizes consumption of plant-based food, olive oil and moderate alcohol intake. Alternative Mediterranean Diet score was used to assess dietary patterns.	Extended Cox proportional hazards models to estimate hazard ratios of mortality in relation to ambient air pollution levels. Fully adjusted multivariable models include the following covariates: age, sex, region, race or ethnic group, level of education, marital status, body mass index, alcohol use, smoking status, and household income.	Long-term exposure to PM_2.5_ had statistically significant associations with cardiovascular disease (CVD), ischemic heart disease (IHD) and cerebrovascular disease. Long term exposure to NO_2_ had statistically significant associations to cardiovascular disease and ischemic heart disease. Participants who had a higher alternative Mediterranean diet score had statistically significant lower rates of CVD and IHD mortality risk associated with long-term air pollution exposure.	The main limitations reported by the authors were the measures of diet were collected a baseline and the study could not account for changes in intake over time, personal covariates were only recorded at baseline and their changes could not be tracked over time, no information on residence location was available for participants that moved out of the region which could result in exposure misclassification, the cohort has a limited number of participants in races other than white and non-black Hispanic and therefore findings may not be generalized to the US population, Mediterranean dietary pattern may also reflect other overall healthy behaviors that were not fully adjusted for in this analysis.
Romieu et al., 2009 [53]	Panel cohort study	Analyze the relationship between diet (fruits, vegetables and Mediterranean diet) and lung function (measured as Forced expiratory volume in 1 second (FEV1) and Forced vital capacity (FVC)) and airway inflammation (IL-8 levels in nasal lavage) after short-term exposure to air pollutants.	Mexico	Children (ages 6 to 14 years old)	208 (158 asthmatics and 50 non-asthmatics)	PM_2.5_, NO_2_, and O_3_ were measured at 4 fixed site central monitors for all days of the study period (505 days). Readings from the closest monitoring station was assigned to the child’s geo-referenced residential address. No fixed site monitoring station was more than 5 km from a child’s home or school.	Dietary assessment to calculate fruit and vegetable intake (FVI) and a Mediterranean diet index (MDI) from mothers’ responses to a 108-item food frequency questionnaire administered at baseline to mothers.	Linear mixed effects models adjusted for gender, body mass index, previous day minimum temperature, corticoid use and chronological time. Other variables such as age, socioeconomic index, outdoor activities, atopic status, exposure to environmental tobacco smoke, use of anti-allergy medicine and season were not significant (*p* > 0.10) and did not alter the results by >1%. Interaction between air pollutant exposure and dietary intake was tested to assess any modifying effect of diet on adverse effect of air pollution on lung health. The effect of the nutritional indices was also assessed in children exposed to low and high levels of pollutants.	In asthmatic children, higher FVI scores were significantly associated with lower IL-8 levels in nasal lavage. In asthmatic children, children in the highest category of MDI had a higher FEV1 and FVC than children in the lowest category and this effect was statistically significant. In non-asthmatic healthy children, there were no statistically significant effects of diet.	The main limitations reported by the authors were the dietary intake food frequency questionnaire was provided by the child’s mother, some error in reporting dietary intake is unavoidable, small sample size, and non-asthmatic children are known to have higher levels of antioxidants in the serum which might have inherently influenced their susceptibility to pollutants.
**Fish Oil and Other Oil Supplements**										
Romieu et al., 2005 [54]	Randomized double blinded trial	Analyze the effect of fish oil versus soy oil supplements on the relationship between particulate matter with a diameter of 2.5 micrometers or smaller (PM_2.5_) exposure and heart rate variability.	Mexico	Adults (nursing home residents, older than 60 years)	50	Daily 24-h measurements of PM_2.5_ measured by using Mini-Vol portable air samplers for indoors of the nursing home, and outdoors at the nursing home site conducted on the roof of the nursing home during the follow-up period.	Randomization to either 2 g/d of fish oil versus 2 g/d of soy oil as the control. Measurements at 1-mo pre-supplementation and 5-mo post-supplementation.	Random-effect regression models adjusted for age, sex, heart rate, body mass index, hypertension. Subsequent models were also adjusted for systolic or diastolic blood pressure and day of the week. Analysis was stratified by hypertension diagnosis.	Reduction in heart rate variability with same day exposures to indoor PM_2.5_ during the pre-supplementation phase. Participants given soybean oil supplements experienced marginal and non-significant protection from the effects of PM_2.5_ on heart rate variability. Supplementation with 2 g/d of fish oil prevented heart rate variability effects related to PM_2.5_ exposure in the study population.	The main limitations reported by the authors were the small sample size, the use of short-term heart rate variability recordings which might have hindered the possibility of performing an adequate assessment on very low frequencies, and the fact that the pre-supplementation phase was limited to 1 month (and consequently the confidence limit around the point estimate for the effect of PM_2.5_ on heart rate variability during this phase was greater)
Romieu et al., 2008 [55]	Randomized, double blinded, controlled trial	Analyze the effect of n-3-polyunsaturated fatty acid (PUFA) supplements (in fish oil) on protection from cardiac alterations linked to PM_2.5_ exposure.	Mexico	Adults (nursing home residents, older than 60 years)	52	Daily 24-h measurements of PM_2.5_ measured by mini-vol portable air samplers (indoor and ambient levels) located in the living room of the nursing home during the follow-up period. O_3_ was monitored by an automated stationary monitoring site located 3 km upwind from the study site.	Randomization of either fish oil (n-3-PUFA) or soy oil supplements (both 2 g/d capsules). Measurements during a pre-supplementation phase of 3 months and a supplementation phase of 4 months.	Linear mixed models which account for repeated measurements within the same individual and allow for variation of effect among individuals were used to determine the effect of PM_2.5_ exposure on biomarkers of in the fish and soy oil supplementation groups. Adjusted for time. Average PM_2.5_ levels were broadly the same between the two treatment periods.	Participants who took the supplementation had greater Cu/Zn superoxide dismutase (SOD) activity, and increased levels of reduced glutathione (GSH) in plasma. The fish oil group experienced statistically significant decreased levels of lipoperoxidation (LPO) products in response to PM_2.5_. These effects were particularly pronounced in the fish oil group compared to the soy oil group.	The main limitations reported by the authors were that exposure assessments were limited to a stationary 24-h gravimetric analysis of PM_2.5_, the small sample size limited the exploration of interactions among supplementation groups and PM_2.5,_ and a larger sample size is required to analyze the lack of significance in the soy oil group.
Lin et al., 2019 [56]	Randomized, double blinded, placebo-controlled trial	Analyze the extent to which fish oil supplements can protect cardiovascular health against PM_2.5_ exposure.	China	Adults (healthy college students between the ages of 20 and 25 years old)	65	PM_2.5_ was measured from fixed sites on campus in real time.	Randomly assigned to either a placebo (sunflower seed oil) or fish oil supplementation (2.5 g per day) for 5 months with 4 rounds of follow up visits with an interval of 2 weeks in the last 2 months of the intervention.	Linear mixed effects models to evaluate PM_2.5_ in relation to cardiovascular health outcomes adjusted for age, sex, body mass index, 3-day average temperature and 3-day average relative humidity.	The placebo group had statistically significantly higher levels of biomarkers of oxidative stress (higher levels of oxidized low-density lipoprotein (ox-LDL) and lower activities of antioxidant capacity), coagulation, endothelial dysfunction (higher levels of endothelin-1 and E-selectin, and lower levels of concentration of serum eNOS protein), neuroendocrine disturbance and blood inflammation than the fish oil group.	The main limitations reported by the authors were that PM_2.5_ exposures were from fixed monitoring sites rather than personal monitors; healthy college age students may be less susceptible to the adverse effects of PM_2.5_ and the benefits of supplementation. Another limitation is cannot exclude the potential confounding effects of daily dietary intakes of omega-3 fatty acids and other nutrients.
Tong et al., 2012 [57]	Randomized, double blinded controlled trial. Controlled exposure to pollutant.	Analyze the extent to which fish oil supplements can mitigate the adverse cardiac effects of exposure to CAP (concentrated ambient PM) and ultrafine particulate matter.	United States	Adults (ages 50 to 72 years old)	29	Controlled exposure to CAP for two hours (mean: 278 ± 19 µg/m^3^, by drawing ambient air from above the roof and passing it through a 2-stage aerosol Harvard concentrator that produces up to a 30-fold increase in particle number and mass)	Randomly assigned to either a fish oil supplementation or an olive oil supplementation (both 3 g/d) for 4 weeks before sequential chamber exposure to filtered air or CAP for 2 h	Two-factor (supplement and PM (particulate matter) concentration) mixed effects model with a subject specific random intercept. Changes in HRV, cardiac repolarization, and blood parameters were assessed at two time points: immediately and approximately 20 h after exposure to CAP and filtered air	Participants taking the fish oil supplement were protected from cardiac electrophysiological and lipid changes after exposure to inhaled CAP while the olive oil group was not. Fish oil supplementation attenuated CAP-induced reductions in high-frequency/low-frequency ratio and normalized low-frequency HRV. Olive oil supplementation group experienced very low-density lipoprotein and triglyceride concentrations.	Olive oil did not provide protection in this study despite the fact it is an important part of the Mediterranean diet. The main limitations reported by the authors were the small number of participants whose results may not be applicable to the population as a whole. Another limitation is related to the fact that the exposure to air and CAP was not randomized.
Tong et al., 2015 [58]	Randomized, double blinded, controlled trial, with controlled exposure to pollutants	Analyze the effect of olive oil and fish oil in mitigating endothelial dysfunction (a mechanism of cardiovascular diseases) and disruption of homeostasis induced by PM exposures.	United States	Adults (ages 50 to 72)	42	2 h exposure to CAP or filtered air (mean: 253 ± 16 µg/m^3^, by drawing ambient air from above the roof and passing it through a 2-stage aerosol Harvard concentrator that produces up to a 30-fold increase in particle number and mass)	Randomization of either 3 g/d of olive oil or 3 g/d of marine derived n-3-fatty acid fish oil or placebo for 28 days (4 weeks), prior to CAP exposure.	Two-factor (supplement and CAP concentration) mixed effects model with a participant specific random intercept. To examine the influence of sex on CAP response and outcomes, sensitivity analysis was conducted by adjusting for sex as a confounder and by restricting analyses to women only.	CAP induced vascular endothelial dysfunction, which was assessed by flow mediated dilation, in middle-aged volunteers who did not receive dietary supplementation. Participants given olive oil (but not fish oil) supplements further experienced a statistically significant reduction in flow mediated dilation and increases in fibrinolysis blood markers in response to the CAP exposure.	Olive oil is a principal component of the Mediterranean diet. Olive oil contains oleic acid which has antioxidant and anti-inflammatory properties. Olive oil should be further investigated as a possible intervention to protect against adverse vascular effects from air pollution exposure. The main limitations reported by the authors were the small number of participants potentially making the results not applicable to the population as a whole. The modest sample size and number of secondary end points measured might have affected the statistical significance of the findings. Another limitation is related to the fact that the exposure to air and CAP was not randomized.
**Sulforaphane**										
Heber et al., 2014 [59]	Randomized, single blinded, placebo-controlled trial. Controlled exposure to pollutant	Analyze the effect of standardized broccoli sprout extract (contains sulforaphane) on the nasal inflammatory response in human subjects after the exposure to 300 µg of DEP.	United States	Healthy adults over the age of 18 and who tested positive for cat allergens via skin prick test	29	Diesel exhaust particles (DEP) administered from a dilution tunnel constant volume sampler. 300 µg in an aqueous suspension (stated as ‘equivalent to daily PM exposure levels on a Los Angeles freeway’.)	Standardized dose of 100 micromolar sulforaphane in mango juice administered daily for 4 days prior to DEP exposure.	For data following a normal distribution, mixed model analysis of variance was used for analysis. The model accounted for the fact that subjects were being compared to themselves. For data that did not follow a normal distribution, medians were reported in addition to means, and non-parametric Wilcoxon signed rank methods were used to compute p-values.	White blood cells in nasal lavage increased after exposure to DEP. When participants consumed the broccoli sprout extract for four days, their white blood cell count in nasal lavage was decreased by 54%.	Small sample size for the study. White blood cell counts in nasal lavage served as a biomarker for inflammatory responses. The main limitations reported by the authors were that the presence of GSTM1 or GSTP1 Ile/Ile105 polymorphisms did not significantly affect the DEP-induced pro-inflammatory effects although there may have been a modulating effect which was not captured due to the small sample study size.
Egner et al., 2014 [60]	Randomized, placebo-controlled trial	Analyze the extent to which daily consumption of the broccoli sprout beverage could detoxify the air pollutants and whether daily doses in a 12-week time frame would make a sustainable difference.	China	Adults (ages 21 to 65 years)	267	24-h averaged concentrations of PM_10_ levels provided by Qidong Environmental Monitoring Station and Shanghai Environmental Monitoring Center, Shanghai Environmental Protection Bureau were recorded during the 84-day study period.	Randomization of a placebo beverage or a broccoli sprout beverage daily for 84 consecutive days (12 weeks).	For the baseline comparison of the treatment and placebo arms, a two-sample t-test of geometric means for each biomarker was conducted. To describe the acute and persistent effects of treatment, separate log linear mixed effects models for each biomarker were fitted.	Participants who received the broccoli sprout beverage had a significantly higher excretion of S-phenyl mercapturic acid (SPMA) and hydroxypropyl mercapturic acid (3-HPMA) both of which are formed in the metabolism of benzene. Increased level of excretion of SPMA and 3-HPMA indicate that the broccoli sprout beverage enhanced the detoxification of pollutants.	SPMA and 3-HPMA are levels of mercapturic acids formed in the metabolism of benzene. The main limitations reported by the authors were modest number of participants (267), 0.3% of the urine samples were not collected, 0.2% of the blood samples were not collected, and participants were under no dietary restrictions during the trial. Other aspects of the participants’ diets were not controlled for.
**Vitamin C and E**										
Sienra-Monge et al., 2004 [61]	Randomized, double blinded, placebo control	Analyze the effect of antioxidant supplements on the nasal inflammatory response that is caused by ozone exposure in atopic asthmatic children.	Mexico	Children with asthma (between the ages of 7 and 11 years old)	117	8-h moving averages of O_3_ and PM_10_ levels obtained from the Mexican government’s air monitoring station, no further than 5 km away from child’s home were recorded during the study period (17 May 1999 to 19 April 2000).	Randomization of either a daily supplement (50 mg vitamin E and 250 mg vitamin C) or a placebo with measurements of nasal lavages taken 3 times during a 4 month follow up and analyzed form content of interleukin-6, IL-8, uric acid and glutathione.	Mixed effect models were used to analyze longitudinal data adjusted for age, exposure to environmental tobacco smoke, atopy, temperature, relative humidity, asthma severity, week of the study, total protein, use of corticoids and uric acid levels in nasal lavage. Regression coefficients were compared using a *t*-test to estimate the effect of the supplementation.	After ozone exposure, children who received the placebo had statistically significant increases in interleukin-6 (IL-6) in nasal lavage while children receiving the supplement did not. Results were similar for interleukin-8 but were not significant. Uric acid decreased slightly in the placebo group.	IL-8 is a mediator of inflammation. Antioxidant supplements, such as vitamin C and E, could decrease the nasal inflammatory response to air pollutants. The main limitations reported by the authors were the exposure assessment being based on a monitoring network and not a personal measurement which could lead to misclassification of exposure, and the fact that the authors did not account for information of allergens indoors and outdoors.

## Abbreviations

ADHD	Attention deficient hyperactivity disorder	HCs	Hydrocarbons
aMED	Alternative Mediterranean diet	HRV	Heart rate variability
BC	Black carbon	IHD	Ischemic heart disease
CAP	Concentrated ambient particulate matter	LPO	Lipoperoxidation
CO	Carbon monoxide	MDI	Mediterranean diet index
COPD	Chronic obstructive pulmonary disease	NO_2_	Nitrogen dioxide
CVD	Cardiovascular disease	NOx	Nitrogen oxides
DEP	Diesel exhaust particles	O_3_	Ozone
EC	Elemental carbon	ox-LDL	oxidized low-density lipoprotein
ECAT	Elemental carbon attributable to traffic	PM	Particulate matter
FDA	Food and Drug Administration	PM_2.5_	Particulate matter with a diameter of 2.5 micrometers or smaller
FEV1	Forced expiratory volume in 1 second	PM_10_	Particulate matter with a diameter of 10 micrometers or smaller
FVC	Forced vital capacity	PUFA	Polyunsaturated fatty acids
FVI	Fruit and vegetable intake	RCT	Randomized controlled trial
GSH	Glutathione	ROS	Reactive oxygen species
TSP	Total suspended particles	SO_2_	Sulfur dioxide
UFPs	Ultra fine particles	SO_4_	Sulfate
VOC	Volatile organic compounds	SOD	Superoxide dismutase
TRAP	Traffic-related air pollution		
